# Postpartum contraception utilization and its impact on inter pregnancy interval among mothers accessing maternity services in the public sector hospital of Hyderabad Sindh

**DOI:** 10.12669/pjms.35.6.914

**Published:** 2019

**Authors:** Seema Bibi, Anila Shoukat, Pir Maroof, Sara Mushraf

**Affiliations:** 1 Prof. Seema Bibi, Head of the Department of Obstetrics and Gynaecology Unit 3, Liaquat University of Medical & Health Sciences, Jamshoro, Sindh, Pakistan; 2 Dr. Aneela Mehmood, Assistant Prof. Department of Obstetrics and Gynaecology, Liaquat University of Medical & Health Sciences, Jamshoro, Sindh, Pakistan; 3 Dr. Pir Maroof, Medical Graduate, Liaquat University of Medical & Health Sciences, Jamshoro, Sindh, Pakistan; 4 Sara Mushraf, 5^th^ Year Student, College of Medicine, Northern Border University Arar, Kingdom of Saudi Arabia

**Keywords:** Interpregnancy interval, Long acting reversible contraception, Post-partum contraception

## Abstract

**Objective:**

To find out the particulars regarding contraceptive practices in the postpartum period and to see its impact on prolonging interpregnancy interval (IPI).

**Methods:**

A cross sectional observational study was conducted in the Department of Obstetrics & Gynecology Unit 3, Liaquat University Hospital Hyderabad from 1^st^ July to 31^st^ December 2018. Out of 2100 total deliveries, 398 postpartum women with second or higher order births were included. Pregnant women, primiparas and those who were not willing to participate were excluded. They were interviewed face to face by principal investigator and her team members and required information was collected on predesigned Performa. Outcome variable was interpregnancy interval in months. Chi-square test was used to see association

**Results:**

Postpartum contraception utilization (PPC) was 24.6%(n=398). Most of the women choose condoms as contraceptive method (n=41, 10.3%) followed by oral contraceptive pills (n=18, 4.5%) and intra uterine contraceptive device (n=16,4%). Method related issues were the chief reasons for discontinuation while desire to have another child soon and spouse/family disapproval were among the reasons mentioned largely by non-utilizers of modern methods of family planning. The average interpregnancy interval was 16.4±1.45. Significant association was observed between interpregnancy interval and postpartum contraception use, particularly in the users of long acting reversible contraceptive methods (P-Value 0.0001).

**Conclusion:**

Uptake and continuation of modern methods of contraception was low after last birth culminating in short interpregnancy interval. Optimal IPI was observed in those utilizing modern methods of family planning, with marked effect in users of long acting reversible contraceptive methods.

## INTRODUCTION

World Health Organization has acknowledged family planning as one of the major interventions to improve the health as well as to save the precious lives of mothers and children worldwide.^1^ Besides its individual health benefits its social and economic benefits for families and communities needs no explanation.^2^ Although effective family planning is important during entire reproductive life of women but contraception utilization during postpartum period is vital as this period holds utmost threat for safety of mother and child.

Postpartum contraception (PPC) is used to prevent unintended and closely spaced pregnancies in the first 12 months after giving birth. Uptake of Post-partum contraception has remarkable effect to prevent unintended pregnancies and improve maternal health. It has the potential to expand inter pregnancy intervals (IPI) and subsequent maternal and obstetrical complications like preterm labour, small for gestational age, low birth weight and maternal anaemia.^3^ Despite high unmet need of family planning after birth, post-partum contraception use is very disappointing. A study conducted in five low income countries including India, Pakistan, Zambia, Kenya and Guatemala revealed an unmet need ranging from 25% to 96% along with very low use of modern contraceptive methods particularly long active reversible contraceptive methods.^4^ Therefore, this study was planned to get recent insight of postpartum contraceptive practices among women accessing maternity services in a very busy and large public tertiary care hospital of Hyderabad Sindh with the primary objective of elaborating the prevalence and details of postpartum contraception utilization including type and duration of method used, reasons for discontinuation and non-utilization. Secondary objective was to find out the association of PPC with inter pregnancy interval. The rationale is to provide data for policy makers, stake holders, scholars and health care providers in planning and implementing policies to boost family planning services in order to improve maternal health and control rapid population expansion.

## METHODS

This cross sectional observational study was carried out in the Department of Obstetrics and Gynaecology Unit 3, Liaquat University Hospital Hyderabad, Pakistan between 1^st^ July to 30^th^ December 2018. Out of 2100 total deliveries, 398 postpartum women with second or higher order births were included. Pregnant women, primiparas and those who were not willing to participate were excluded. They were interviewed face to face by principal investigator and her team members and required information was collected on predesigned Performa, after taking verbal informed consent. Data included socio-demographic details, obstetrical history, inter pregnancy interval in months, and details of postpartum contraception use including method, duration and reasons for discontinuation and non-utilization.

Inter pregnancy interval (IPI) defined as period between delivery date of preceding birth and conception date of index pregnancy. It is calculated by interval between two consecutive deliveries minus gestational age of second infant(neonate). It is calculated in weeks and converted into months. For this study, IPI was categorized into three groups i.e. >24 months (optimal),12 to 24 months(short) and <12 months (very short). Contraceptive methods were also grouped into long acting reversible contraception (LARC), short acting reversible contraception (SARC) and barrier methods.

Simple random technique was used for data collection. Sample size of 398 was used taking the percentage of current contraception utilization as 35%^5^. Statistical analysis was performed using SPSS version 10.0. Results were expressed as number and percentages. Duration of inter pregnancy interval in groups was compared with groups of contraceptive methods. Chi square test (Fisher exact) test was used to see association between PPC and inter pregnancy interval. P-value of less than 0.05 was considered significant.

The study approval was granted by ethical review committee of the Liaquat University of Medical and Health Sciences Jamshoro (Ref. No. LUMHS/REC/-788).

## RESULTS

Demographic and obstetrical details were shown in Table-I. The Mean± SD of age and parity of the respondents was 28.4 ± (4.9) years and 4.0 (±3.16) respectively. Majority were uneducated (74.9%, n=298) and residents of urban area (70.6%, n=281). The average inter pregnancy interval (IPI) was 16.4±1.45 with the majority (82%, n=326) having interpregnancy interval of less than 24 months.

**Table I:** Socio-demographic details among the studied women, (N=398).

Variable	Number	Percent (%)
*Maternal age group (years)*		
< 19	21	5.3
19 – 40	375	94.2
> 40	2	5
Mean (± SD)	28.4 (±4.9)	
Parity		
< 2	141	35.4
2- 4	142	35.7
> 4	115	28.9
Mean (± SD)	4.0(±3.16)	
*Educational level*		
Uneducated	298	74.9
Primary	51	12.8
Matric	35	8.8
Inter	12	3.0
Graduate	2	0.6
*Husband Education*		
Uneducated	232	58.3
Primary	73	18.3
Matric	43	10.8
Inter	42	10.6
Graduate	8	2.1
*Inter pregnancy period (months)*		
<12	201	50.5
12-24	125	31.4
>24	72	18.1
Mean (± SD)	16.4(1.45)	
Residence		
Rural	117	29.4
Urban	281	70.6

Particulars of postpartum contraception utilization among study participants are shown in Table-II. Overall Postpartum contraception utilization was 24.6%(n=398) and most of them stopped using before 24 months and only 10%(n=39) continued for more than 24 months. Most of the women choose condoms as contraceptive method (10.3%, n=41) followed by oral contraceptive pills (4.5%, n=18) and intra uterine contraceptive device (4%, n=16). Table-II shows that method related factors (side effects, poor compliance, non-availability) were the chief reasons for discontinuation of modern methods of family planning. While desire to have another child soon and spouse/family disapproval were among the reasons mentioned largely by non-utilizers (21 -23%) as compared to women who discontinued the method (5%).

**Table II:** Details of PPC utilization among the studied women. (n=398).

Variables	Number	Percentage %
***1. Postpartum contraception utilization***		
Yes	98	24.6
No	300	75.4
***2. Type of method used***		
(a) Long acting reversible contraception (LARC)	31	7.8
i.Intra uterine contraceptive device (IUCD)	16	4.0
ii.Injections	13	3.26
iii.Implants	02	0.5
(b) Short acting reversible contraception (SARC)	26	6.5
i.Oral contraceptive pills (OCP)	18	4.52
ii.Emergency contraceptive pills (ECP)	08	2.0
(c) Barrier method(Condoms)	41	10.3
***3. Duration of PPC utilization(months)***		
(a) < 12	12	3
(b) 12 to 24	47	12
(c) >24	39	10
***4. Reasons for discontinuation of PPC***		
(a) Method related problems (side effects, poor compliance, non-availability)	37	9.3
(b) No reason mentioned	23	5.8
(c) Desire to conceive again	19	4.8
(d) Spouse/family disapproval	19	4.8
***5. Reasons for non-utilization of PPC***		
(a) No reason mentioned	90	23
(b) Desires to conceive soon.	90	23
(c) Spouse/family disapproval	83	21
(d) Lack of knowledge of methods, supplies and fear of side effects	37	9.2

Association of inter-pregnancy interval with duration of postpartum contraception and type of method used are shown in Table-III. Significant association was observed between utilization of postpartum contraception and inter pregnancy interval (P-value 0.0001). Optimal inter pregnancy interval of more than 24 months was observed among women using long acting reversible contraceptive methods as compared to short acting reversible contraception(SARC) and barrier methods (P-value 0.0001).

**Table III:** Association of PPC with Inter pregnancy interval.

Variables	IPI <12 months	IPI 12 to 24 months	IPI >24 months	Total	P-value
1.PPC utilization	12	47	39	98	0.0001
2.Type of method:					0.0001
Long acting reversible contraception (LARC)	0	10(21.3%)	21(53.8%)	31(31.6%)
Short acting reversible contraception (SARC)	3(25%)	13(27.7%)	10(25.6%)	26(26.5%)
Barrier method	9(75%)	24(51.1%)	8(20.5%)	41(41.8%)

## DISCUSSION

A recent Demographic and Health Surveys data from 21 low and middle income countries including Pakistan revealed high unmet need of family planning in postpartum period with overall 31% utilization of family planning during postpartum period. Reported post-partum family planning utilization among Pakistani women was 26% which is in line with results of our study.^6^ In contrast, very low usage of PPC i.e. 4% is reported from the neighboring city of Thatta, Sindh. This gross dissimilarity may be attributed to data collection site as our study is urban based from tertiary care hospital in comparison to community based data of rural Thatta.^4^ Place of residence has long been considered as prognosticator of PPC use.^7^,^8^ Besides the differences in preferred family size, lack of accessibility and availability of preferred methods of contraception significantly affects the family planning practices of rural women as compared there to their urban counterparts.^9^,^10^

A very distressing fact noted in this study is that majority of users discontinue contraception before 24 months. Method related problems like occurrence of side effects, poor compliance and lack of availability were among the important reasons for discontinuation. Our figures are coherent with recent population council report from Pakistan which revealed that more than a third of women discontinue contraceptive method within a year due to a method related reasons, including side effects, method failure dissatisfaction and lack of access and affordability.^10^ In comparison to south Asian women of India and Bangladesh, method related problems were reported to be main reason for Pakistani women to stop contraception. Naz and Mahmoud while highlighting this fact suggest that eradicating method related hitches will likely to increase continuation rates by 6 to 10%.^11^ Simple course of actions like proper counseling at start of modern contraceptive methods, timely and appropriate management of common side effects seems to be cost effective and worth trying strategies for Pakistani health care providers.

Among the non-utilizers of post-partum contraception, disapproval from spouse or family members and desire to have another baby soon were the key reasons. These facts point towards the strong patriarchal family organization in Pakistan where women’s reproductive autonomy and decision making is lacking. A large family size with more male siblings is still highly valued despite monetary challenges of nurturing large families. However, a recent study from Punjab Pakistan found changing trends where males wanted fewer children and willing to take technical information about family planning.^12^ Use of condoms by notable number of the study participants suggest that male involvement is an overlooked intervention which can be effectively utilized by recognizing strategies based on situation analysis approach.

Inter-pregnancy interval (IPI) is defined as the time intervened between two consecutive pregnancies and duration of 24 months is considered to be optimal.^13^ Moorea Z et al. during an analysis of 22 surveys from low and middle-income countries found that short inter pregnancy interval occurred in 40 to 55% cases with highest proportion in Pakistan.^6^ Evidence suggest that Postpartum women who did not use contraceptives or delay in the use of contraceptives after birth were least likely to be sheltered against pregnancy culminating in shorter IPI.^6^,^14^ In line with various studies, our study showed an alarming situation as 81.9% (326) of participants have IPI of less than 24 months. However, optimistic side of the picture is significant association of IPI interval with PPC utilization. Another worth noted figure is strong impact of type of contraceptive method with IPI. Majority of study participants who opted for LARC (IUD, Injections, implants) have optimal duration of IPI as compared to short acting reversible contraceptives (OCP, ECP) and barrier (condoms) methods. An association which has been witnessed in women belonging to both developed and under developed countries.^6^,^15^

Although LARC methods were found to be enhancing IPI interval but regrettably negligible number of study participants were found to be using these methods. Most of the reasons mentioned by study participants for non-uptake of modern methods are in line with the barriers identified by other researchers like dearth of proper knowledge, method related issues and supply chain challenges.^16^,^17^ According to population council report about 55 to 69% Pakistani women who used Injections and IUD(LARC) stopped due to insufficient information about side effects, their management and choice of switching to other method.^10^ In order to improve compliance and reduce method related discontinuation certain simple interventions will be helpful. The first step is proper evaluation of women’s health, her reproductive wishes and sociocultural background. Second step is to help the women to choose the method which is best for her in terms of efficacy, safety, ease to use, accessibility and availability. Central to all is involving women and their partners in decision making, addressing their fears and misconception about side effects, management of side effects and options of switching to other methods. Targeted media campaigns utilizing TV, radio, newspapers and social media focusing women, men and other family members will help in changing the mindsets of community.

Supply chain system failure was also one of the reported obstacles affecting availability of modern family planning methods in low and middle income countries.^17^ This fact which is also disclosed in this study. Poor infrastructure, deficient logistics and dearth of trained and motivated staff are well-known explanations for supply chain failures. Interventions aimed towards improving infrastructure, ensuring sustained contraceptive supplies along with the availability of qualified and skilled staff will likely to improve existing situation.^18^,^19^ Role of community health workers in the promotion of modern family planning methods is well established. In Pakistan, Lady Health Workers (LHW) can be actively engaged to promote PPC uptake by focusing women during peripartum period. They can be trained and supervised to offer frequent home visits for effective counselling and provision of modern FP methods. An approach that has been found fruitful in Chad, Congo and Bangladesh.^8^,^19^

### Strength and limitations of the study

One of the important limitations is its observational study design and random technique of data collection imperiling to information and selection bias. The second limitation is comparatively small sample size in relation to huge public health problem related to family planning. The strength of study is data collection from 1450 bed public sector tertiary care hospital having massive obstetrical load and large catchment area. Results can be generalized as study participants are representing large population from urban, sub urban and rural areas of Sindh and neighboring province of Balochistan and Punjab.^20^

## CONCLUSION

Small number of study participants used contraception after last birth, mostly relying on condoms and short acting hormonal methods culminating in early discontinuation of contraceptive methods and short interpregnancy interval. The study found that desire to conceive again soon and disapproval of spouse or family members were main causes for not starting any method after last birth while majority of users stopped due method related factors. Uptake of long active reversible contraceptives expands interpregnancy interval but were found to be least utilized methods. Improving awareness in the society about importance of family planning is the key to enhance post-partum contraceptive practice. High discontinuation rates demand for improving family planning services, mainly counselling, to help women for informed choice, prepare them about possible side effects and their management including method switching. Ensuring accessibility and sustained supply will further improve the existing situation. Male partner involvement is crucial for breaking socio-cultural as well as technical barriers.

### Authors’ Contribution:

**SB** designed the study, performed the analyses and drafted the manuscript, takes responsibility for integrity of research.

**AM** helped in data collection and analysis.

**SM** assisted in analysis and drafting.

**PM** reviewed the final draft.

All authors read and approved the final version of the manuscript.

## References

[1] World Health Organization (2018). Family planning/Contraception key fact sheet. Geneva.

[2] Sonfield A, Hasstedt K, Kavanaugh ML, Anderson R (2013). The Social and Economic Benefits of Women's Ability to Determine Whether and When to Have Children.

[3] Conde-Agudelo A, Belizan JM (2000). Maternal morbidity and mortality associated with interpregnancy interval:Cross Sectional Study. BMJ.

[4] Pasha O, Goudar SS, Patel A, Garces A, Esamai F, Chomba E (2015). Postpartum contraceptive use and unmet need for family planning in five low-income countries. Reprod Health.

[5] National Institute of Population Studies (NIPS) [Pakistan] and ICF International. 2013 Pakistan Demographic and Health Survey2012-2013.

[6] Moorea Z, Pfitzer A, Gubinb R, Charuratb E, Elliottc L, Crofta T (2015). Missed opportunities for family planning:an analysis of pregnancy risk and contraceptive method use among postpartum women in 21 low- and middle-income countries. Contraception.

[7] Eliason S, Bockarie A, Eliason C (2018). Postpartum fertility behaviors and contraceptive use among women in rural Ghana. Contracept Reprod Med.

[8] Kamal Mostafa SM, Rahman A, Anisur M, Towhiduzzaman (2017). The Influence of Family Planning Workers on Postpartum Contraceptive use among Women in Bangladesh. J Fam Med Commun Health.

[9] Ouma S, Turyasima M, Acca H, Nabbale F, Obita KO, Rama M (2015). Obstacles to family planning use among rural women in Atiak Health Center IV, Amuru District, Northern Uganda. East Afr Med J.

[10] Zaidi B, Hussain S Reasons of Low Modern Contraceptive Use - Insights from Pakistan and other Neighboring Countries 2015. Population Council, Pakistan.

[11] Mahmood A, Naz SS Contraceptive Use dynamics in Pakistan 2008-09. March 2012. Population Council, Islamabad.

[12] Iram K, Khan M, Zeba T (2014). Involving men in reproductive and fertility issues:insights from Punjab (English). Health, Nutrition, and Population (HNP) discussion paper.

[13] Conde-Agudelo A, Rosas-Bermudez A, Kafury-Goeta AC (2006). Birth spacing and risk of adverse perinatal outcomes:A meta-analysis. JAMA.

[14] Eliason SK, Bockarie AS, Eliason C (2018). Postpartum fertility behaviours and contraceptive use among women in rural Ghana. Contracept Reprod Med.

[15] Thiel de Bocanegra H, Chang R, Howell M, Darney P (2014). Interpregnancy intervals:impact of postpartum contraceptive effectiveness and coverage. Am J Obstet Gynecol.

[16] Jabeen C, Umbreen G (2016). Long Acting Reversible Contraceptives:Knowledge and Attitude among Married Women of Lahore. J Liaquat Uni Med Health Sci.

[17] Mukasa B, Ali M, Farron M, Van de Weerdtd R (2017). Contraception supply chain challenges:a review of evidence from low- and middle-income countries. Euro J Contracept Reprod Health Care.

[18] United Nations Population Fund UNFPA Supplies Annual Report 2015.

[19] Rattan J, Noznesky E, Curry DW, Galavotti C, Hwang S, Rodrigueza M (2016). Rapid Contraceptive Uptake and Changing Method Mix With High Use of Long-Acting Reversible Contraceptives in Crisis-Affected Populations in Chad and the Democratic Republic of the Congo. Glob Health Sci Pract.

[20] Liaquat University Hospital Hyderabad.

